# O31 Giant cell arteritis - a rare ocular presentation

**DOI:** 10.1093/rap/rkab067.030

**Published:** 2021-10-19

**Authors:** Priti Kulkarni, Shireen Shaffu, Arumugam Moorthy, Bharat Kapoor, Periyasamy Kumar

**Affiliations:** 1 Ophthalmology department, Leicester Royal Infirmary, Leicester, United Kingdom; 2 Rheumatology department, Leicester Royal Infirmary, Leicester, United Kingdom

## Abstract

**Case report - Introduction:**

Giant cell arteritis (GCA) is a systemic vasculitis primarily affecting large- and medium-sized arteries. Classic symptoms include headache, scalp tenderness, jaw claudication and visual disturbances.

Ophthalmic artery involvement commonly causes anterior ischaemic optic neuropathy. Uncommon ocular features include anterior segment ischaemia, hypotony, tonic pupil or rarely choroidal ischaemia. Heterogenicity of presentation can make diagnosis difficult leading irreversible visual loss.

We report a case of bilateral macular choroidal ischaemia with atypical symptoms of GCA. It emphasises the need of complete evaluation in elderly patients with GCA and visual symptoms and the need to start aggressive treatment to prevent visual loss.

**Case report - Case description:**

73-year-old caucasian lady presented to the eye emergency department with diplopia. Medical history includes systemic hypertension, hypothyroidism and hyperlipidaemia, no past ocular history. Eye examination was normal except decompensated fourth nerve paresis. Thyroid function was normal. Diplopia resolved spontaneously.

Patient re-presented with a floater in the right eye and left-sided atypical headache without jaw claudication. Investigations: normal FBC, CRP 126, ESR 100, PV 1.67. Following rheumatologist review she was commenced on oral prednisolone 60 mg with clinical suspicion of GCA. Temporal artery biopsy confirmed GCA. She had TIA subsequently. MRI revealed small area of acute infarct in left ganglio-capsular region. Clopidogrel was started for secondary prevention.

In the ophthalmology clinic she saw a lacy pattern. Her Log MAR VA in right and left eye was 0.64 and 0.76, respectively. Fundoscopy revealed retinal pigment epithelial (RPE) mottling at the maculae, right more than left eye. Optical Coherence Tomography (OCT) macula revealed bilateral RPE elevations and serous pigment epithelial detachment bilaterally, patchy central RPE atrophy with external limiting membrane disruption, more pronounced in the right eye. Fundus fluorescein angiogram and indocyanine green angiography confirmed bilateral choroidal ischaemia (triangular shaped with the base at the equator) at the macula worse in right than left eye.

Oral prednisolone was continued with gradual tapering. VA improved to Log MAR 0.5 and Log MAR 0.2 in right and left eye at six weeks. OCT showed signs of RPE re-modelling with resolution of sub retinal fluid (resolution of inflammation).

At recent follow up Log MAR VA is 0.26 and 0.06 in right and left eye respectively. She is on oral prednisolone 20 mg once a day tapering 2.5 mg every 2 weeks. OCT shows further re-modelling of the ellipsoid zone in the left eye, but her right eye shows more RPE atrophy and thinning with RPE degeneration.

**Case report - Discussion:**

We report an unusual case of GCA with atypical symptoms and bilateral choroidal ischaemia.

Patients with GCA usually present with systemic symptoms and signs like headache, scalp tenderness, fever, and jaw claudication. Variable presentation can often lead to misdiagnosis and consequent irreversible loss of vision. Visual symptoms as the first and only sign of GCA was first reported in 1952.

Posterior ciliary arteries in the eye can be affected leading to optic nerve infarction and subsequent anterior ischaemic optic neuropathy (AION). AION and visual field loss accounts for 80—90% of cases with ocular signs of GCA. Posterior ciliary artery occlusion can rarely cause patches of choroidal infarcts which appear as chorio-retinal degeneration in a couple of weeks. These patches are usually in the mid-peripheral fundus, usually triangular shaped with the base towards equator and apex toward posterior pole.

In our case the presentation was very atypical in the sequence of symptoms. Her raised inflammatory markers raised the suspicion of GCA and prompt referral to rheumatology was done. Aggressive treatment with oral steroids was started with stomach and bone protection. Temporal artery biopsy confirmed the diagnosis. The bilateral triangular ischaemic areas found on FFA and ICG confirmed the macular choroidal ischemia. Her OCT also showed bilateral RPE mottling showing degenerative changes due to choroidal infarct from posterior ciliary artery occlusion.

We managed to preserve the vision in our case by starting the timely aggressive steroid treatment.

In summary, we report an unusual case of GCA with atypical symptoms and bilateral choroidal ischaemia where further visual loss was avoided due to timely intervention. GCA has variety of presentations; a combined team approach of ophthalmologists and rheumatologists can prevent irreversible visual loss in such cases.

**Case report - Key learning points:**

GCA is a chronic idiopathic inflammation more commonly seen in the large- and medium-sized vessels.

Posterior ciliary arteries in the eye can be affected in GCA leading to optic nerve infarction and subsequent anterior ischaemic optic neuropathy (AION). AION and visual field loss accounts for 80—90% of cases with ocular signs of GCA. Posterior ciliary artery occlusion can rarely cause patches of choroidal infarcts which appear as chorio-retinal degeneration in a couple of weeks. These patches are usually in the mid-peripheral fundus, usually triangular shaped with the base towards equator and apex toward posterior pole.

Prompt diagnosis and aggressive treatment with corticosteroids can prevent visual loss in one or both eyes. Any patient over 50 years of age presenting with visual symptoms of amaurosis fugax, diplopia, or visual loss with ocular signs of anterior or posterior ischaemic optic neuropathy, central retinal artery occlusion or cilioretinal artery occlusion should create a high suspicion for GCA. This group of patients should have urgent ESR, CRP and PV evaluation. If suspected, high-dose corticosteroids must be started followed by temporal artery biopsy for confirmation. It is imperative to diagnose GCA early and start treatment urgently to prevent visual loss.

A multidisciplinary team approach in patients with GCA can prevent sight loss and life too.

